# Antitumour responses induced by a cell-based Reovirus vaccine in murine lung and melanoma models

**DOI:** 10.1186/s12885-016-2536-2

**Published:** 2016-07-13

**Authors:** Ciorsdan A. Campion, Declan Soden, Patrick F. Forde

**Affiliations:** Cork Cancer Research Centre, Western Gateway Building, University College Cork, Cork, Ireland; School of Microbiology, University College Cork, Cork, Ireland

**Keywords:** Reovirus, Immune, Cytokine, Cancer Vaccine

## Abstract

**Background:**

The ever increasing knowledge in the areas of cell biology, the immune system and the mechanisms of cancer are allowing a new phase of immunotherapy to develop. The aim of cancer vaccination is to activate the host immune system and some success has been observed particularly in the use of the BCG vaccine for bladder cancer as an immunostimulant. Reovirus, an orphan virus, has proven itself as an oncolytic virus in vitro and in vivo. Over 80 % of tumour cell lines have been found to be susceptible to Reovirus infection and it is currently in phase III clinical trials. It has been shown to induce immune responses to tumours with very low toxicities.

**Methods:**

In this study, Reovirus was examined in two main approaches in vivo, in mice, using the melanoma B16F10 and Lewis Lung Carcinoma (LLC) models. Initially, mice were treated intratumourally (IT) with Reovirus and the immune responses determined by cytokine analysis. Mice were also vaccinated using a cell-based Reovirus vaccine and subsequently exposed to a tumourigenic dose of cells (B16F10 or LLC). Using the same cell-based Reovirus vaccine, established tumours were treated and subsequent immune responses and virus retrieval investigated.

**Results:**

Upregulation of several cytokines was observed following treatment and replication-competent virus was also retrieved from treated tumours. Varying levels of cytokine upregulation were observed and no replication-competent virus was retrieved in vaccine-treated mice. Prolongation of survival and delayed tumour growth were observed in all models and an immune response to Reovirus, either using Reovirus alone or a cell-based vaccine was also observed in all mice.

**Conclusion:**

This study provides evidence of immune response to tumours using a cell-based Reovirus vaccine in both tumour models investigated, B16F10 and LLC, cytokine induction was observed with prolongation of survival in almost all cases which may suggest a new method for using Reovirus in the clinic.

## Background

Rapidly growing, aggressive tumours continue to be a major challenge to current treatment protocols. Both melanoma and lung carcinomas often demonstrate poor prognosis and metastasise rapidly making therapy even more of a challenge [1]. The American Cancer Society estimates that 135,000 people in the US are diagnosed annually with melanoma and approximately 50 % of these will be invasive at the time of diagnosis. While melanoma is by no means the most common skin cancer diagnosed, it is responsible for the most deaths [2]. Over 50 % of lung cancers have already metastasised at the time of diagnosis and 5-year survival at this stage is less than 5 %. Overall, lung cancers are responsible for 20 % of all cancer deaths worldwide (WHO) [3]. Models reflecting these two aggressive tumour types were investigated in this study.

Traditionally, oncolytic viruses are self-replicating and tumour selective viruses which will not produce disease in the host [4]. Respiratory Enteric Orphan Virus or Reovirus is a non-pathogenic virus initially isolated from human respiratory and gastrointestinal tracts in the 1950s [5, 6]. Reovirus is not associated with any known disease and has been shown to induce apoptosis in cells carrying a mutation in a certain pathway, the Ras pathway, which is found in a wide variety of tumour types [7, 8]. In vitro, over 80 % of cell lines of tumour origin tested have been found to be susceptible to Reovirus-related oncolysis [9].

Reovirus as a cancer therapy was approved for clinical trials by the FDA as Reolysin® with the first phase I study conducted in 2002 in patients with a variety of malignancies [10]. Several phase II and III studies have since been conducted in a wide range of malignancies, investigating Reovirus alone and in combination with current treatment modalities [11, 12]. The majority of trials currently underway are investigating the combination of Reolysin® with existing chemotherapy agents such as carboplatin and paclitaxel [13].

Cancer vaccination is an area of cancer therapy that has been in development for many years with several approaches being developed. The aim of vaccination in cancer therapy is to activate the host immune system. The most straightforward method of vaccine development is to select antigens that are expressed on the tumour – tumour associated antigens – such as p53 [14].

Oncolytic viruses are ideal candidates in the role of cancer vaccination due to their tumour-selective nature and the means by which they induce cell death provides a natural selection of tumour-associated antigens (TAAs) [15, 16]. Several strategies have been or are currently being investigated in an effort to amplify the cancer vaccination role of oncolytic viruses including genetic manipulation to express additional proteins such as Heat Shock Proteins (HSPs), cytokines and chemokines. Clinically, a poxvirus expressing GM-CSF (Pexa-Vec) demonstrated antitumour immunity in several phase I/II trials and was also shown to be capable of inducing antibody-mediated cancer cell lysis. Similarly, adenovirus expressing several HSPs proved to be capable of inducing tumour antigen-specific CD8+ T cell responses in melanoma and colorectal cancer [17–21]. Figove et. al, have previously investigated the potential of Reovirus in a vaccine role in a HPV16 transformed murine model with some delay in tumour development observed however survival was not reported [22].

In this study, mouse models were administered a cell-based Reovirus vaccine as a prophylaxis vaccine and their ability to withstand subsequent challenges observed. This cell-based Reovirus vaccine was also investigated as a therapeutic vaccine in the murine models B16F10 and Lewis Lung Carcinoma (LLC). Immune responses were also explored as the role of the immune system becomes ever more important in the effective treatment of cancer [23].

## Methods

### Virus propagation and quantification

Reovirus Type 3 Dearing (T3D) was obtained from The National Collection of Pathogenic Viruses (Public Health England). Virus was propagated and plaque assayed on L929 cells (mouse fibroblast). Confluent flasks of L929 were infected with Reovirus T3D with the virus allowed to adsorb for approximately 1h at 37 °C in a humidified atmosphere containing 5 % CO_2_ in air. Following incubation the viral inoculum was removed and fresh medium added and cells were incubated for 48h. The medium was then collected and cell debris removed by centrifugation (140g for 10mins).

This clarified supernatant was then plaque assayed to determine the viral titre. 6-well plates were seeded with L929 cells and allowed to become 100 % confluent. 10-fold serial dilutions of the virus were performed and 400μl of each dilution were plated in duplicate in wells of 6 well plates containing the L929 monolayers. The inoculum was allowed to adsorb for 1h at 37 °C in a humidified atmosphere containing 5 % CO_2_. Following adsorption, the inoculum was removed and 2ml of overlay medium, 1:1 2 % agar:2xMinimum Essential Medium (MEM) containing 0.8 % Foetal Calf Serum, 0.4 % L-glutamine and 0.2 % penicillin/streptomycin, were added to well. The overlayed monolayers were allowed stand at room temperature until the overlay had solidified. The plates were incubated at 37 °C in a humidified atmosphere containing 5 % CO_2_ for 72h. Following 72h incubation, the monolayers were fixed with 10 % formylsaline (formaldehyde in phosphate buffered saline (PBS)) for 45 min to 1h at room temperature. The agar overlay was then carefully removed and the monolayers stained with 2 % crystal violet (crystal violet in 96 % ethanol) for 10 mins. The monolayers were then gently washed in water. Plaques appear as clear areas on a purple background.

### Cell culture

The tumour cell lines B16F10 and Lewis Lung Carcinoma (LLC) were obtained from the American Type Cell Collection (Manassas, VA). Both cell lines were grown in Dulbecco’s Modified Eagle’s Medium (DMEM) (Sigma Aldrich) supplemented with 10 % v/v Foetal Calf Serum (Gibco) and 1 % v/v 20X L-glutamine (Sigma Aldrich). Cells were maintained at 37°C in a humidified atmosphere containing 5 % CO_2_ in air.

### Animals and tumour induction

For both B16F10 and LLC experiments, C57Bl/6j mice (Harlan Laboratories, UK), approximately 6–8 weeks old were used. The mice were kept in a pathogen free environment for at least one week prior to experiments. For routine tumour inoculation, cells were harvested from tissue culture flasks by trypsinisation and counted using a haemocytometer. For tumour induction of B16F10 tumours, 5 × 10^5^ cells were resuspended in 200μl of serum-free DMEM. LLC tumours were induced using 2 × 10^5^ cells resuspended in 200μl of serum-free DMEM. Tumour inoculation was performed subcutaneously (s.c.) into the right flank. Following establishment, the tumours were measured using vernier callipers, in two dimensions. Tumour volume was calculated using the formula V = ab^2^π/6 where a is the longest diameter of the tumour and b is the longest diameter perpendicular to a. Mice were humanely euthanized by cervical dislocation when tumour volume reached 1.5cm^2^.

### Virus purification

For administration of Reovirus intratumourally, virus was initially pelleted using ultracentrifugation. Appropriate aliquots of virus were centrifuged at 28,400 × g for 1h using Beckman Coulter Polyallomer centrifuge tubes. The virus pellets were then resuspended in serum-free DMEM.

### Cell-based Reovirus vaccine

B16F10 and LLC cells were infected at an MOI of 10 PFU/cell. Following an adsorption period of 1 h, the virus inoculum was removed, fresh medium added and the cells incubated overnight at 37 °C. After overnight incubation, the cells were trypsinised, washed twice with PBS and counted. For B16F10 based vaccine, 5 × 10^6^ cells were considered a vaccine dose while 2 × 10^6^ cells were used for LLC vaccination. Viable cells were present in each vaccine dose as determined by colony formation assay (data not shown).

### Virus administration and vaccination regimen

Resuspended virus for IT administration was injected IT in an escalating dose regimen over a three week period. Two doses of virus were administered in every treatment week.

Week 1; 1 × 10^7^ PFU; Week 2; 1 × 10^8^ PFU and Week 3; 1 × 10^9^ PFU

**Fig. 1:**
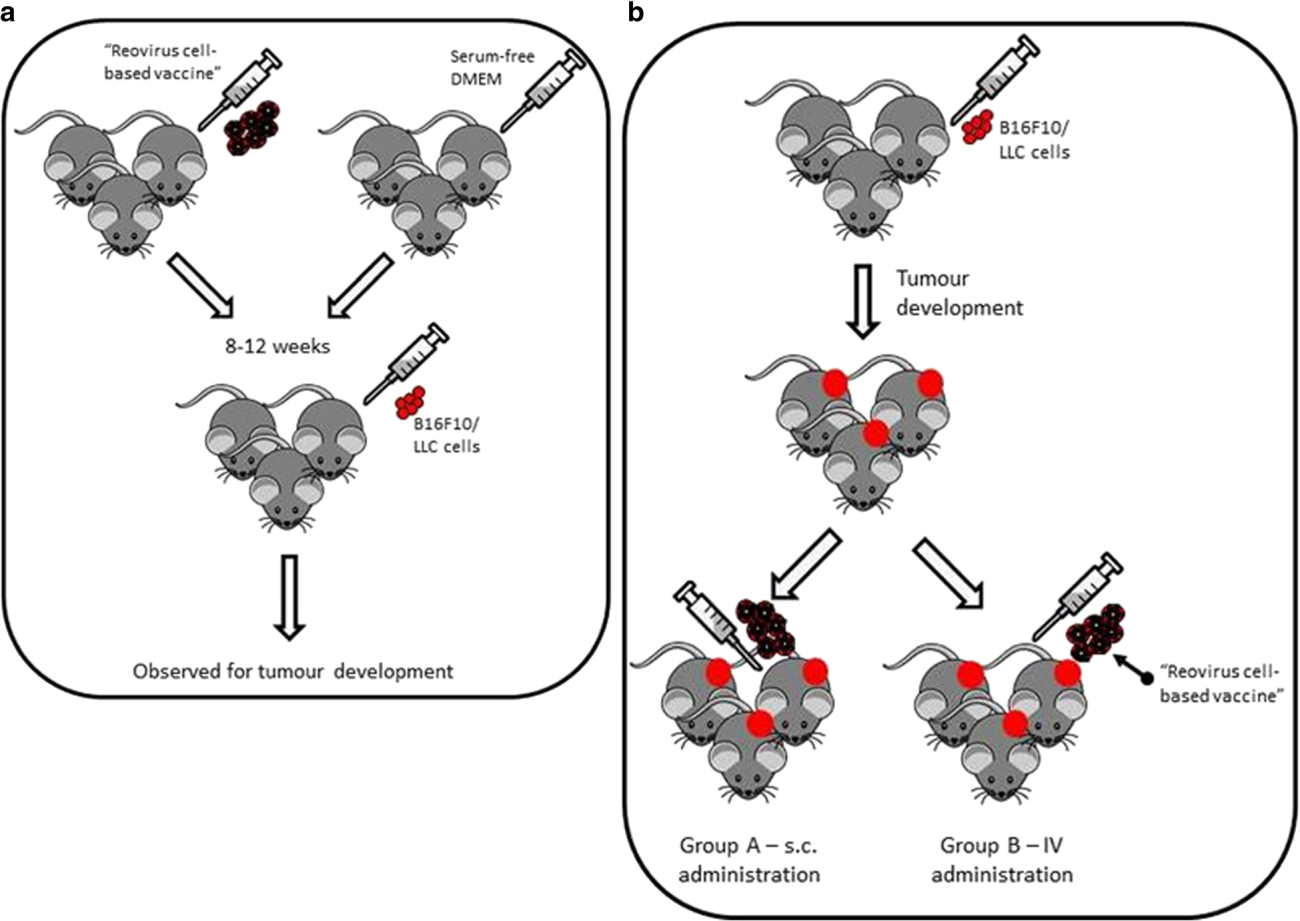
Schematic representation of the vaccination regime. **a** Regime of cell-based Reovirus vaccine as a prophylactic and (**b**) as a therapeutic for established tumours

A cell based vaccination was administered by two main approaches; as a prophylactic vaccine and as a therapy for established tumours (Fig. 1). For prophylaxis, naïve mice were inoculated s.c. with the vaccine as prepared above. 8–12 weeks following vaccination, mice were inoculated with a tumourigenic dose of the appropriate cell line (B16F10 or LLC). Tumour volume was measured as indicated previously. For a therapeutic approach, tumours were induced in mice as indicated previously in C57Bl/6j mice. When tumours became palpable, treatment was administered by means of a vaccination s.c. on the opposite flank to the tumour or intravenously (IV) via the tail vein. Tumour volume was measured as indicated.

### In vitro cytotoxicity assay and adoptive transfer of splenocytes

Splenocytes were harvested from mice previously vaccinated and challenged and from naïve mice. 2 × 10^6^ splenocytes were incubated with 2 × 10^5^ mitomycin-C treated target cells in the presence of 25IU/ml IL-2 with both sets of experiments (vaccinated and naïve) being performed in triplicate. The splenocyte/cell mixtures were incubated for 5 days at 37 °C in a humidified atmosphere containing 5 % CO_2_ in air and following incubation the lymphoid cells were harvested. The cells were washed three times with serum-free DMEM, to ensure any remaining lymphoid cells were removed, and applied as effectors in effector:target ratios of 20:1 and 1:1 with 2×10^4^ target cells. The cells were incubated overnight in round bottomed 96 well plates. Wells were washed five times with PBS and an MTT assay was performed to quantify remaining living cells. 200μl of MTT solution (2mg/ml in serum-free DMEM) were added to each well. The plate was then incubated at 37 °C in a humidified atmosphere containing 5 % CO_2_ in air for 4h. Following incubation, the MTT was removed by aspiration and 200μl of DMSO added to each well. The plate was shaken for 2–3 min and the absorbance for each well at OD570nm was measured.

Immune-mediated anti-tumour responses following vaccination were investigated using a modified Winn assay. Splenocytes were harvested from mice previously vaccinated and challenged and naïve mice. Splenocytes were then mixed with the target cell (B16F10/LLC) in a ratio of 50:1. Following 48h incubation, the splenocyte/cell mixture was injected s.c. into naïve mice at a tumourigenic dose for each particular cell line. Mice were monitored for tumour development

### Cytokine analysis

Excised tumours were lysed by homogenisation in RIPA buffer (Sigma Aldrich) (with proteinase inhibitors). Lysates were then tested on a multiplex mouse Th1/Th2 multi-spot assay as per manufacturer’s protocol (MesoScale Discovery). Briefly, the level of protein was normalised using a Bio-Rad protein assay (data not shown) and a protein level of 50–100μg/25μl was used. 25μl of each lysate was plated in triplicate and incubated overnight at 4 °C with shaking. The wells were then washed three times in PBS-0.05 % Tween 20. 25μl of detection antibody was added to each well and the plate was incubated at room temperature for 2h with shaking. The wells were washed three times in PBS-0.05 % Tween 20 and 150μl of read buffer was added to each well. The plate was then read on a MesoScale SECTOR imager. Reagents; antibody, read buffer and calibrators were made up according to manufacturer’s instructions.

### Plaque assay of tumour samples

Tumours were excised from mice that had received either the cell-based Reovirus vaccine or purified virus as therapy. Samples were weighed, homogenised and centrifuged to obtain a clear supernatant. Cell supernatants were collected and plaque assayed on L929s as described previously and expressed as PFU/mg of tissue.

### Statistical analysis

Experimental results were plotted and analysed for significance with Prism 5 software (GraphPad software Inc. CA, USA). *P* < 0.05 was considered significant. Error bars are representative of (mean + SEM).

## Results

### Intratumoural treatment of established tumours with Reovirus alone results in a reduction of tumour burden and a moderate extension on survival

**Fig. 2:**
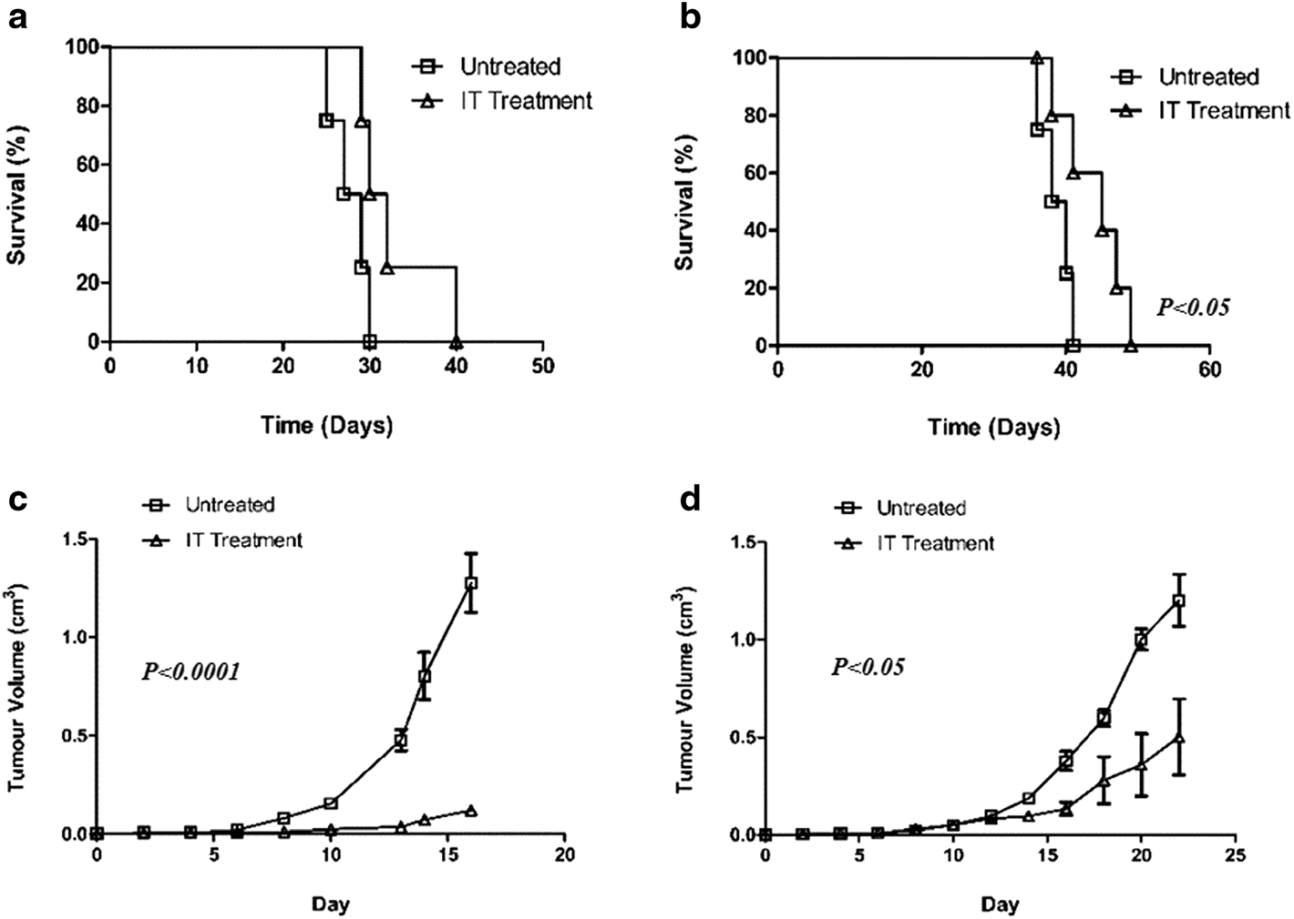
Treatment of established B16F10 and LLC tumours with purified Reovirus administered IT. **a** Survival and (**c**) tumour volume of B16F10 tumours in C57Bl/6j mice (*n* = 5) following treatment with Reovirus in an escalating dose regimen. **b** Survival and (**d**) tumour volume of LLC tumours following the same regimen in C57Bl/6j mice (*n* = 5)

Mice (*n* = 5) received purified Reovirus IT in an escalating dose regimen, twice weekly for three weeks from 1 × 10^7^ PFU to 1 × 10^9^ PFU. A statistically significant increase in survival was observed in Lewis lung carcinoma-bearing (LLC) mice (*P* < 0.05) (Fig. 2b). A moderate increase was observed in B16F10-bearing mice (*n* = 5) as compared to untreated mice (Fig. 2a). Tumour volume of both LLC and B16F10 showed that there was a delay in the development of tumours particularly B16F10 following IT treatment (Fig. 2c, d). Mice received IT Reovirus as soon as tumours became palpable, B16F10 tumour bearing mice showed a statistically significant slower rate of tumour growth compared to untreated mice (*P* < 0.0001). LLC tumour-bearing mice also showed a significantly slower rate of tumour growth in treated mice compared to untreated (*P* < 0.05).

### Safety and efficacy of a cell-based Reovirus vaccine

**Fig. 3:**
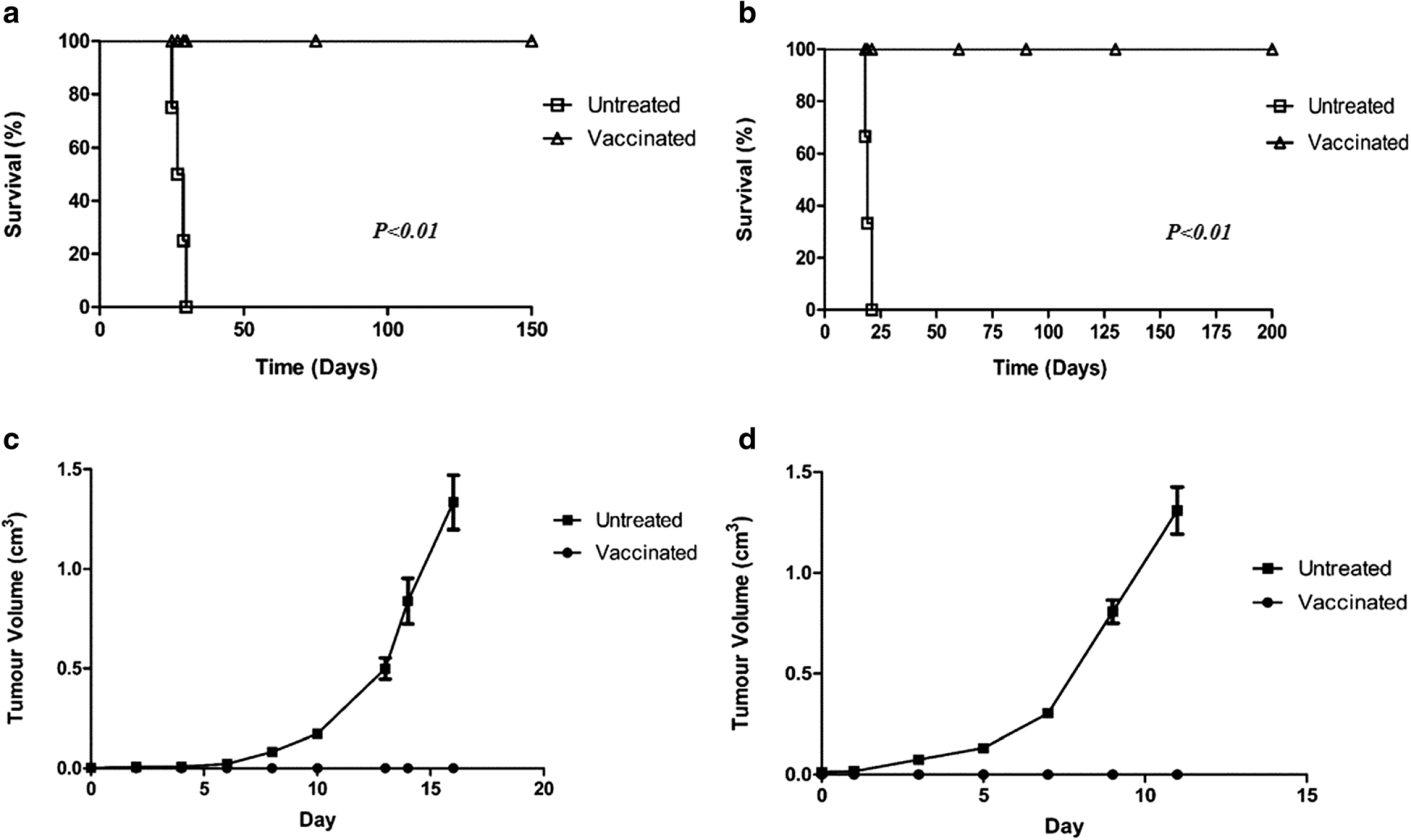
Challenge of “vaccinated” mice. For both groups (B16F10 and LLC), mice received a cell-based Reovirus vaccine 8–12 weeks prior to day 0. Survival of (**a**) B16F10 and (**b**) LLC vaccinated mice compared to naïve controls. In both incidences vaccinated mice survived beyond 100 days which was deemed to be curative. Unvaccinated mice developed tumours within 10 days for B16F10 inoculated mice (**c**) and 7 days for LLC inoculated (**d**)

Two groups of mice (for each cell line) were inoculated with a tumourigenic dose of cells. The first group of C57BL/6J mice had previously received a cell-based Reovirus vaccine 8–12 weeks prior to challenge (either B16F10 vaccine or LLC vaccine as appropriate). No tumour development was observed in vaccinated mice following vaccine administration, B16F10 or LLC. The second group were naïve mice i.e. unvaccinated. Median survival of B16F10 injected naive mice was deemed to be 28 days (*n* = 5). No tumours were observed in mice that had previously been “vaccinated” prior to challenge and survival was beyond 150 days which was deemed to be curative (*P* < 0.01) (Fig. 3a). A similar trend was seen in LLC injected mice (*n* = 5) where the median survival of unvaccinated mice was 19 days while vaccinated mice survived beyond 150 days (*P* < 0.01) (Fig. 3b). *** To test the specificity of the vaccine received, mice vaccinated against B16F10 were inoculated with a tumourigenic dose of LLC and vice versa. In both instances, tumours developed and survival was similar to that of unvaccinated and challenged mice (data not shown).

### In vitro cytotoxicity and adoptive transfer of lymphocytes

**Fig. 4:**
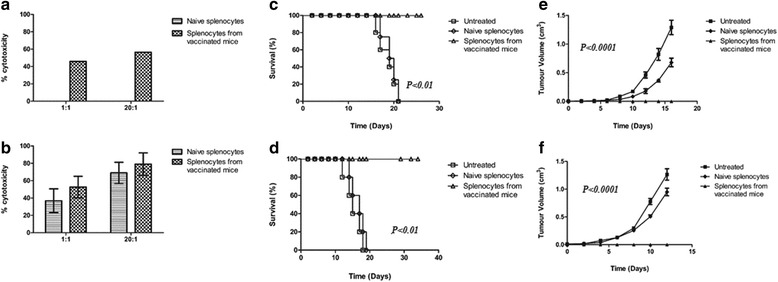
Cytotoxicity of splenocytes isolated from vaccinated mice. Splenocytes were obtained either from naïve mice or from mice that had been previously vaccinated (either B16F10 or LLC as appropriate). **a** and (**b**) In vitro cytotoxicity of B16F10 and LLC splenocytes from naïve mice and vaccinated mice against the target cell (B16F10 or LLC as appropriate). Modified Winn assay to determine if the cell-based vaccine could induce long-term immunity in vivo for B16F10 (**c, e**) and LLC (**d, f**). Inoculation groups consisted of (1) target cells (B16F10 or LLC) (2) splenocytes from naïve mice mixed with target cells and (3) splenocytes from vaccinated mice mixed with target cells

Splenic T lymphocytes obtained from mice vaccinated with a B16F10-based Reovirus vaccine had increased cytolytic activity compared to splenocytes obtained from naïve mice as demonstrated using an MTT-based assay (Fig. 4a). Cytotoxicity of LLC vaccinated splenocytes was marginally increased as compared to naïve splenocytes (Fig. 4b). This increased cytotoxicity for both B16F10 and LLC corresponds to the immunity seen in vivo, as demonstrated by a modified Winn assay. Groups of mice (*n* = 5) received a mixture of B16F10 cells and either splenocytes from naïve mice or from vaccinated mice, injected s.c. This was repeated in another group of mice with LLC cells. Both groups of mice that had received vaccinated splenocytes from vaccinated mice (B16F10 & LLC) survived beyond 100 days suggesting a specific immunity generated by vaccination (Fig. 4c, d)(*P* < 0.01). All mice inoculated with splenocytes from naïve mice developed tumours, both B16F10 and LLC, whereas no tumours were observed in mice that had received splenocytes from vaccinated mice for either B16F10 and LLC (Fig. 4e, f) (*P* < 0.0001).

### A cell-based vaccine provides a moderate extension on survival when used as therapy for established tumours

**Fig. 5:**
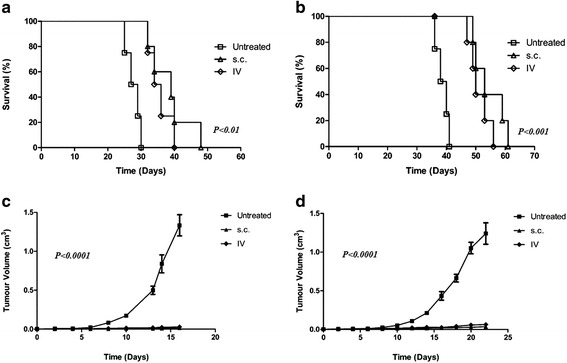
Cell-based Reovirus vaccine for the treatment of established tumours. Survival of (**a**) B16F10 and (**b**) LLC-bearing mice subsequent to treatment with a single dose of cell-based vaccine. Tumour volume of same groups, (**c**) B16F10 and (**d**) LLC, showing a significant delay in tumour development in treated mice

A significant amount of research has been aimed at establishing a vaccine for cancer or cancer-causing agents with some success. However, the majority of therapy is still concerned with eradicating established tumours [24, 25]. To investigate if a cell-based Reovirus vaccine was suitable as a therapy for established tumours groups of mice (*n* = 5) received a tumourigenic dose of B16F10 cells and a tumour was allowed to develop. The mice were subsequently inoculated with a Reovirus B16F10-based vaccine either intravenously (IV) through the tail vein or s.c. Untreated B16F10 bearing mice had a median survival of 28 days as compared to mice that had received therapy which had a median survival of 35 days. Prolonged survival was seen in mice that had received the vaccine s.c. with a median survival of 39 days (Fig. 5a) (*P* < 0.01). This was reflected in the volume of the tumours where untreated mice had an average tumour volume of 1.3cm^3^ while mice treated with a cell-based Reovirus vaccine had an average tumour volume of 0.02cm^3^ and 0.01cm^3^ for IV and s.c. inoculation respectively (Fig. 5c) (*P* < 0.0001). A similar trend was seen with LLC-tumour bearing mice (*n* = 5) with mice receiving treatment s.c. demonstrating a moderate increase in survival, 53 days, compared to the IV group, 50 days. Both were a significant increase in comparison to the untreated group which had a median survival of 39 days (Fig. 5b) (*P* < 0.001). Tumour volumes of “vaccine” treated LLC groups were reflective of the increased survival with the IV group having an average tumour volume of 0.06cm^3^ and the s.c. group, 0.03cm^3^ compared to 1.2cm^3^ of the control group (Fig. 5d) (*P* < 0.0001).

### A cell-based Reovirus vaccine is capable of stimulating an immune response in the tumour microenvironment

**Fig. 6:**
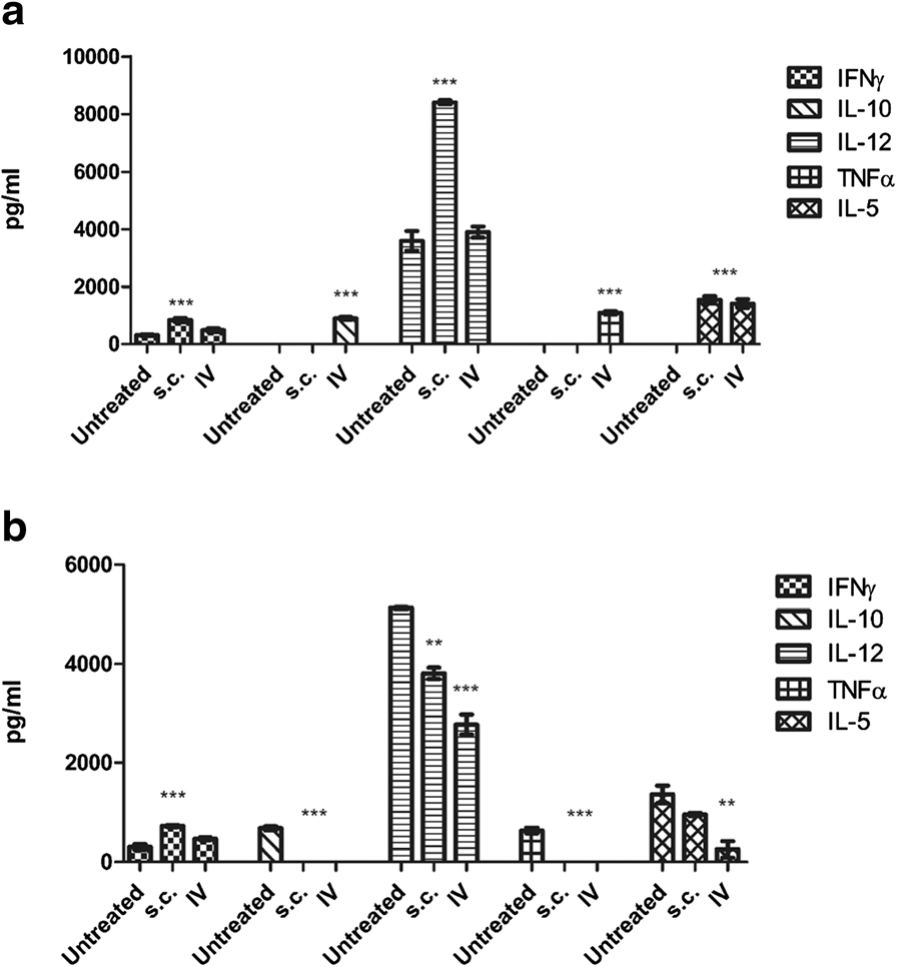
B16F10 and LLC tumours that had previously received cell-based Reovirus vaccine as therapy for established tumour were excised and analysed for cytokine production. Both groups were evaluated – those that had received the vaccine s.c. and IV. **a** B16F10 tumours showed no particular pattern of cytokine production post exposure relative to route of vaccine administration. **b** LLC tumours showed a down-regulation of cytokine levels in almost all evaluated. Statistical significance is indicated by the stars above each bar (*P < 0.05, **P < 0.01, ***P < 0.0001)

The extent of cytokine stimulation or suppression was evaluated in homogenised tumour samples obtained from groups of mice that had previously received a cell-based Reovirus vaccine either IV or s.c. to treat established tumours in both B16F10 and LLC models. Neither mode of administration route appeared to be more favourable with stimulation varying between the cytokine involved and the origin of the tumour. Subcutaneous administration in B16F10 tumour-mice appeared to have a slightly improved effect in terms of cytokine stimulation (Fig. 6a). Interferon-γ, Interleukin-12 and Interleukin-1β, all T helper cell associated cytokines, demonstrated increased production compared to untreated tumours and those that received treatment intravenously. Levels of IL-10 were increased in IV-treated mice compared to s.c. treated; ideally levels of IL-10 should be suppressed by a successful therapy as regulatory T cells in the tumour environment often produce IL-10 as a means of suppressing the immune response to tumour cells. Conversely, IL-10 levels were suppressed in LLC tumour bearing mice by both s.c. administration and IV. Overall, however LLC tumours showed a significant dampening of cytokine stimulation following treatment with both IL-12, a significant cytokine in the immune response to transformed cells and a mediator between the innate and adaptive immune system, and TNFα levels dropping significantly following treatment, both s.c. and IV (Fig. 6b).

### IT administration of purified Reovirus does not demonstrate an increased cytokine reaction compared to a cell-based Reovirus vaccine

**Fig. 7:**
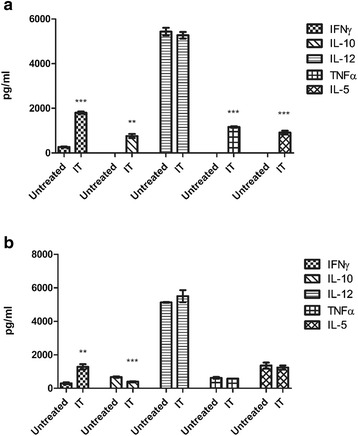
B16F10 and LLC tumours that had previously received purified Reovirus IT were also evaluated for cytokine production by ELISA. B16F10 (**a**) showed a general trend of up-regulation of cytokine production while no significant changes were observed in LLC tumours (**b**) with the exception of IFNγ

Cytokine analysis of tumours, both B16F10 and LLC, that had received Reovirus IT as a means of therapy for established tumours, did not show a markedly different level of stimulation or suppression of cytokines as compared to tumours treated with a cell-based Reovirus vaccine. Levels of IL-12 remained unchanged in both LLC and B16F10 tumours although B16F10 tumours showed stimulation of all other cytokines evaluated while LLC only showed increased levels of IFNγ and IL-1β compared to untreated controls. LLC tumours did display a suppression of IL-10 following treatment with the virus compared to untreated tumours this suppression of IL-10 was not observed in B16F10 tumours (Fig. 7a, b).

### Virus recovery is not indicative of efficacy of treatment

**Fig. 8:**
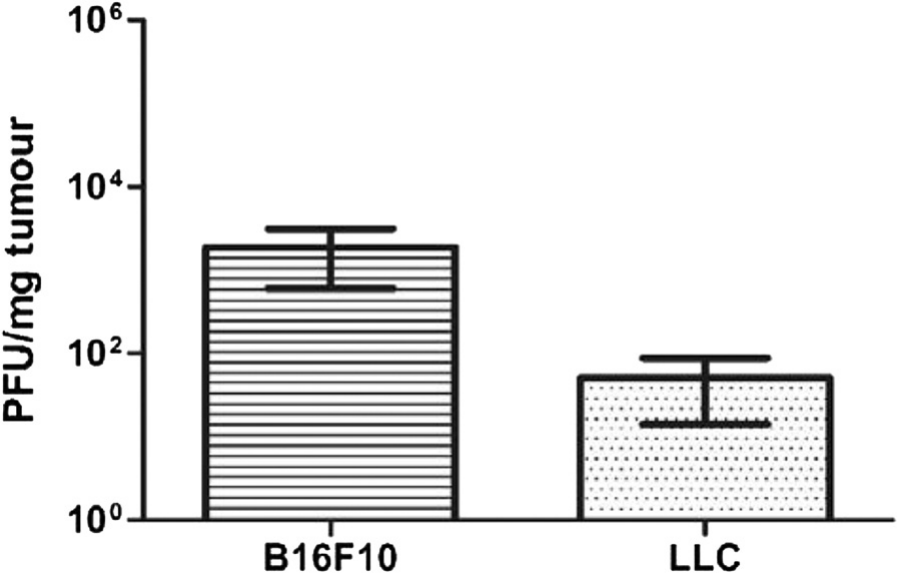
Level of active virus retrieved from treated tumours approximately one week following cessation of treatment. Active virus was retrieved from both B16F10 tumours and LLC tumours that had received purified Reovirus IT. No active virus was retrieved from tumours treated with the cell-based Reovirus vaccine, either B16F10 or LLC

One of the major subjects of debate in virus therapy is the importance of the presence of replication-competent virus in the tumour and its environment. Many studies have indicated that virus titre is not directly proportionate to apoptosis although lack of recovery of active virus has also been blamed for poor response to viral therapy [26]. Both LLC and B16F10 tumours that had received an escalating dose regimen of purified virus IT displayed a low level of virus recovery following treatment (Fig. 8). No virus was recovered from tumours that had received a cell-based Reovirus vaccine as therapy however this group (vaccine group) showed better survival rates than those that received virus IT.

## Discussion

Cancer vaccination has been the subject of much investigation as discussed previously. Approaches include a prophylactic vaccine such as the development of a vaccine against Human Papillomavirus (HPV) to prevent cervical cancer (Gardasil®) or the use of vaccines as therapy for established tumours e.g. the BCG vaccine for bladder cancer therapy [27]. Both these approaches, prophylactic and as a therapy for established tumours, were investigated using a cell-based Reovirus vaccine.

Initial studies of a cell-based Reovirus vaccine showed great success in the treatment of tumours derived from both cell lines evaluated; B16F10 and LLC. Animals challenged with a tumorigenic dose following only one vaccination showed inhibition of tumour growth and all animals survived beyond 100 days. A subsequent modified Winn assay of splenocytes from vaccinated animals showed they were capable of preventing tumour establishment in naïve mice. These results imply that there is long term immunity to the specific tumour cells involved. While these results are promising, it raises the question of the feasibility of the use of prophylactic vaccines, except in rare and specific circumstances. The vast majority of cancers arise spontaneously hence their prediction is impossible although certain populations may benefit such as those predisposed to certain cancers e.g. women who carry the defective BRCA1 or BRCA2 genes which predispose to breast cancer [28]. However they may be applicable in an effort to prevent metastatic disease which was not evaluated in this study. Removal of the primary tumour and subsequent use of a vaccine of this nature, specific for the patient, may be a feasible approach.

The use of the same vaccine in a therapeutic setting in the treatment of established tumours also showed some efficacy with increased rates of survival in all groups. Therapy by means of the vaccine also demonstrated significant delay in the growth of tumours derived from the cell lines. Subcutaneous administration proved marginally more effective than IV. Administration via the subcutaneous route would potentially allow the virally infected cells more exposure to antigen presenting cells (APCs) such as macrophages and dendritic cells which would not be present in the blood (IV administration), stimulating the response to Reovirus.

Interestingly, no replication competent virus was retrieved from any of the tumours treated with the cell-based vaccine. Treatment with the vaccine proved to be more effective in terms of both survival and tumour volume than tumours treated IT with Reovirus alone despite the retrieval of virus from IT treated tumours. It is difficult to determine if the titre of virus in the tumour is of major importance with reports conflicting on the significance of high replication levels and its relation to apoptosis induction. Certainly, the results seen in this study imply that high titres of replication-competent virus are not necessary for effective therapy and likewise, increased titres do not translate to survival benefit.

The immune system plays a major role in cancer by its initial response in an effort to eliminate tumour cells and its subsequent failure which allows cancer to progress [23, 29]. Both B16F10 and LLC tumours exposed to Reovirus alone showed up-regulation of several favourable cytokines including Interferon-γ (IFN-γ) and IL-5 although the cytokines observed and the level of up-regulation varied between B16F10 and LLC. A significant increase in the level of IFN-γ was seen in both lines. IFN-γ has a major role in the activation of macrophages, cytotoxic T cells and NK cells and is positive in terms of immune responses to the presence of tumours. IL-1, a pro-inflammatory cytokine, was also found to be upregulated in both B16F10 and LLC.

B16F10 tumours treated IT also showed increased levels of TNFα. TNFα is currently used in the clinic as immunotherapy [30] and its upregulation leads to the activation of both macrophages and lymphocytes. The activation of these cells (macrophages and B cells) may indicate that both the innate and adaptive immune response are responsive to the presence of Reovirus although interestingly IL-10 was also increased in B16F10 tumours. IL-10 is often used by tumour cells to suppress the immune response in order to escape detection [31]. Its presence may indicate that the tumour is making an effort to down regulate the immune response triggered by the virus either by producing IL-10 directly or inducing infiltrating immune cells to do so.

IT treated LLC tumours showed a reduction in the level of IL-10 which should be a favourable outcome however this did not translate into increased survival. Few cytokines were substantially up-regulated in LLC compared to B16F10 with the exception of IL-2 which was not observed in B16F10 but was noted in LLC (data not shown) which may have contributed to the variance in survival rates. IL-2 is another common cytokine in clinical immunotherapy although its efficacy has been the subject of much debate. Immunologically, IL-2 triggers T cell proliferation and activates Natural Killer (NK) cells [23, 32].

A cytokine panel of B16F10 tumours exposed to the cell-based Reovirus vaccine implies that the mode of administration may influence the responses of the immune system. IV treatment appears to favour cell-mediated responses with T cell proliferation and lymphocyte and NK cell activation induced by the cytokines expressed. S.c. administration favours more inflammatory responses – IFNγ. In contrast, LLC tumours, regardless of mode of administration did not show significantly increased levels of any cytokines evaluated. Despite this, the survival of both LLC groups – IV and s.c. was significantly increased compared to B16F10. Other factors, not investigated here such as tumour lysis syndrome, may be responsible for the differences observed.

A more extended panel of cytokines or other immunological factors may reveal other influences. The impressive ability of tumour cells to transform and to influence the immune system is still a major obstacle in effective therapy [27] however the responses observed in this study are promising for the advancement of Reovirus in the clinic and opens the door for alternative approaches to virus administration.

## Conclusion

This study demonstrates that the use of a cell-based Reovirus vaccine is effective in survival prolongation and immune stimulation and could potentially lead the way for new advancements in cancer immunotherapy.

## Abbreviations

BCG, Bacillus Calmette-Guerin; BRCA1/2, breast cancer ½; DMEM, Dulbecco’s Modified Eagle’s Medium; DMSO, dimethyl sulfoxide; IFNγ, interferon-γ; IL-1/1β/2/5/8/10/12, Interleukin-1/1β/2/5/8/10/12; IT, intratumourally; IU, international units; IV, intravenously; LLC, Lewis Lung Carcinoma; MOI, multiplicity of infection; MTT, (3-(4, 5-dimethylthiazolyl-2)-2, 5-diphenyltetrazolium bromide); NK cells, natural killer cells; PBS, phosphate buffered saline; PFU, plaque forming units; RIPA buffer, radioimmunoprecipitation assay buffer; s.c., Subcutaneous; Th1/Th2, T helper 1/T helper 2; TNFα, tumour necrosis factor-α
